# Nitrate of Potash in Spasmodic Asthma

**Published:** 1844-07-27

**Authors:** 


NITRATE OF POTASH IN SPASMODIC ASTHMA.

Dr. Frisi states, in the Filiatre Sebezio, that having read of a proposal by an American physician to employ, in cases of spasmodic asthma, the vapor of nitrate of potash obtained by burning in the chamber, or smoking in a pipe, porous paper twice soaked in a solution of that substance, he determined to give it a trial in an obstinate case of that disease, which had resisted every other means. The relief was instantaneous, and the remedy whenever repeated, never failed to cut short the attack.—*London Med. Times.*

